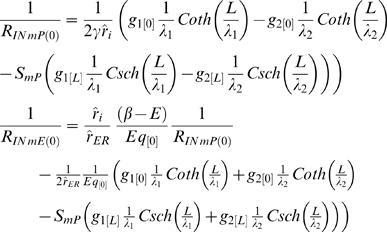# Electrotonic Signals along Intracellular Membranes May Interconnect Dendritic Spines and Nucleus

**DOI:** 10.1371/journal.pcbi.1000036

**Published:** 2008-03-28

**Authors:** Isaac Shemer, Björn Brinne, Jesper Tegnér, Sten Grillner

**Affiliations:** 1 Department of Neuroscience, Karolinska Institutet, Stockholm, Sweden; 2 Division of Computational Biology, Department of Physics, Chemistry, and Biology, The Institute of Technology, Linköping University, Linköping, Sweden; 3 The Computational Medicine Group, Center for Molecular Medicine, Department of Medicine, Karolinska Institutet, Karolinska University Hospital, Stockholm, Sweden; 4 Unit of Computational Medicine, Center for Genomics and Bioinformatics, Karolinska Institutet, Stockholm, Sweden; University College London, United Kingdom

## Abstract

Synapses on dendritic spines of pyramidal neurons show a remarkable ability to induce phosphorylation of transcription factors at the nuclear level with a short latency, incompatible with a diffusion process from the dendritic spines to the nucleus. To account for these findings, we formulated a novel extension of the classical cable theory by considering the fact that the endoplasmic reticulum (ER) is an effective charge separator, forming an intrinsic compartment that extends from the spine to the nuclear membrane. We use realistic parameters to show that an electrotonic signal may be transmitted along the ER from the dendritic spines to the nucleus. We found that this type of signal transduction can additionally account for the remarkable ability of the cell nucleus to differentiate between depolarizing synaptic signals that originate from the dendritic spines and back-propagating action potentials. This study considers a novel computational role for dendritic spines, and sheds new light on how spines and ER may jointly create an additional level of processing within the single neuron.

## Author Summary

Our study incorporates the fact that the endoplasmic reticulum (ER) forms a complete continuum from the spine head to the nuclear envelope and suggests that electrical current flow in a neuron may be better described by a cable-within-a-cable system, where synaptic current flows simultaneously in the medium between the cell membrane and the ER, and within the ER (the internal cable). Our paper provides a novel extension to the classical cable theory (namely, cable-within-cable theory) and presents several interesting predictions. We show that some of these predictions are supported by recent experiments, whereas the principal hypothesis may shed new light on some puzzling observations related to signaling from synapse-to-nucleus. Overall, we show that intracellular-level electrophysiology may introduce principles that appear counter-intuitive with views originating from conventional cellular-level electrophysiology.

## Introduction

Glutamatergic synaptic inputs onto dendritic spines of pyramidal neurons induce phosphorylation of the transcription factor CREB (cAMP-Responsive-Element Binding protein) in the nucleus [1]–[3]. CREB phosphorylation is important for converting specific synaptic inputs into long-term memory in several animal species [4],[5]. Interestingly, action potential (AP) trains induced post-synaptically by direct intracellular current injection fail to initiate CREB phosphorylation [1],[6]. Several studies [1]–[3],[7],[8] have aimed at finding the spine-to-nucleus signaling involved in CREB phosphorylation that enables the nucleus to discriminate between orthodromic and antidromic signals. The nature of this signal transduction, however, remained unclear.

It has been shown that bulk elevation in cytosolic Ca^2+^ is neither necessary nor sufficient for activity-dependent CREB phosphorylation [1],[2],[7]. It was further shown that regenerative Ca^2+^ waves along the dendritic endoplasmic-reticulum (ER) are not necessary for mediating this synapse-to-nucleus signaling [1]. The means by which signals travel from spines to nucleus has therefore been suggested to involve diffusion of a second messenger. Since the Ca^2+^-Calmodulin complex (Ca^2+^/CaM) is readily generated in the spine during synaptic activity and since activity-dependent CREB phosphorylation follows translocation of Ca^2+^/CaM from cytosol to nucleus, Ca^2+^/CaM diffusion was suggested to carry the spine-to-nucleus signal [3],[8]. However, CREB phosphorylation appears 15 seconds after the beginning of the stimulus, which is substantially faster than expected from diffusion of CaM [8]. During a 15 second period, the mean-square displacement of CaM is 5 µm, whereas the diameter of pyramidal somata ranges between 15–20 µm and the most proximal spines do not appear within 10–15 µm from the soma (spine density approaches zero at the first 25 µm, [9],[10] and the first spine was reported to appear 39.7±12.1 µm from the soma [9]). Mermelstein *et al.* have therefore suggested that CaM diffuses in a phosphorylated form, which can reach 20 µm during 15 seconds due to an increased cytoplasmic diffusion rate.

This suggestion, which provides the best, up-to-date, estimate for synapse-to-nucleus signaling, disregards the fact that the spine neck acts as a diffusion barrier for second messengers as small as cAMP, cGMP, and IP3 [11],[12] (molecular weights 300–1000 D; Compared with 16.8 kD [13] for CaM). We hereby suggest an alternative means of signal transduction that readily complies with the described time frame of spine-to-nucleus signaling, namely, an electrotonic signal along *internal* membranes (Table 1 [8], [14]–[16]).

**Table 1:** Expected Traveling Time of Different Means of Signaling. (Calculated for 40 µm synapse-to-nucleus distance, which correspond to ∼30* µm synapse-to-soma distance).

Means	Speed	Time	References
Transport vesicles	11–21 µm/hr	2–4 hr	[14]
Transport granules**	122 µm/hr	20 min	[15]
Diffusion of CaM	D = 2.5×10^−9^ cm^2^/s	48 min	[8]
Diffusion of kinase-bound CaM	D = 5×10^−8^ cm^2^/s	2.7 min	[8]
Regenerative Calcium waves***	∼0.1 µm/ms	0.4 sec	[16]
Electrotonic signal	81 µm/ms	0.5 ms	****

*: The distance from the soma to the most proximal spine was reported to be 39.7±12.1 µm [9].

**: Average maximal speed of granules traveling along a dendrite is 0.034±0.025 µm/s [15].

***: Regenerative Calcium waves were ruled out as means for synapse-to-CREB signaling [1].

****: Electrotonic speed (θ) was calculated according to the parameters in Table 2 using the conventional definition 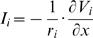 given by the classic cable theory.

By the end of the 90's it was acknowledged that the endoplasmic reticulum (ER) forms a continuous network of tubes and sacs that extends from the nuclear envelope out to the cell periphery [17]–[20]. This view followed studies which employed EM reconstruction [18] and diffusion of dye along internal membranes [20] to show the ER continuity across the axon, soma, dendrites and the spine apparatus at the dendritic spines' heads. Accordingly, the ER has been suggested to act as a ‘neuron-within-neuron’, as originally suggested by Michael Berridge [21]. However, until now signal propagation and integration along the ER have been described to take place via regenerative Ca^2+^ wave [21].

**Figure 1:**
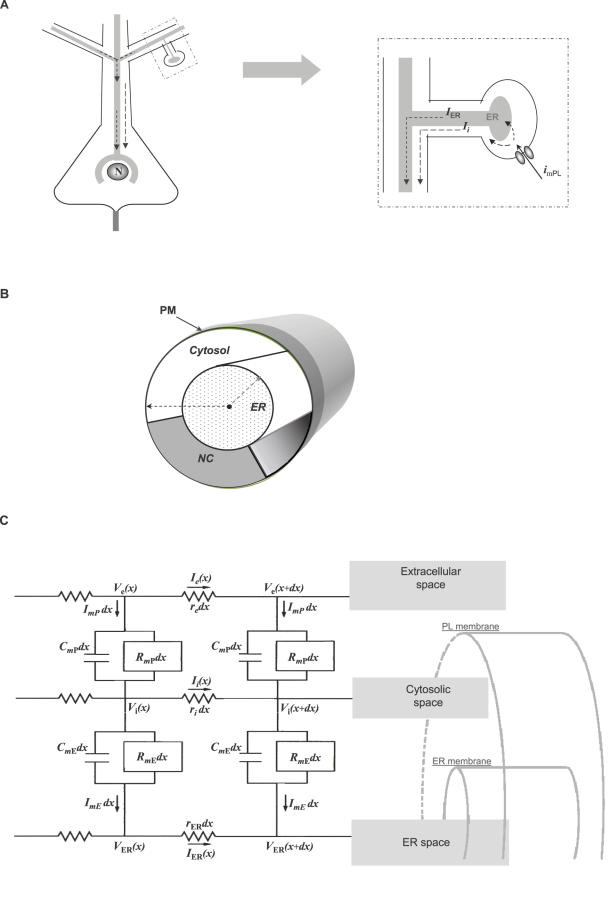
Cable-in-cable hypothesis. (A) The principal hypothesis: synaptic activation onto a dendritic spine, generates two simultaneous currents, along the cytoplasmic compartment and along the ER lumen. These currents generate two passive electrotonic signals, across the plasma membrane (outer cable) and across the ER membrane (the inner cable). Consequently, synaptic activity at the spine can signal electrotonically-fast to the nucleus. (B) The model describes a passive system of a cable within cable where the internal cable represents the ER-lumen confined by ER-membrane and a cytosolic compartment surrounded by plasma membrane. The model also assumes a non-conductive cross section representing various intracellular organelles. (C) The cable-in-cable model provides an analytical solution for a system constructed of iso-potential circuit elements of length *d*x. The circuit describes three semi-infinite compartments: external (*V*_e_), cytosolic (*V*_i_), and ER (*V*_ER_), which are separated by two membranes, represented by a resistor (*R*_m_) and a capacitor (*C*_m_) in parallel. The circuit further defines the positive direction of currents. A similar two layer circuit has been employed previously for modeling non-passive signaling along myelinated axon [69].

Here, we propose a passive electrotonic potential along the ER lumen and across the ER membrane (Figure 1A). This hypothesis is supported by reconstruction studies of spiny dendrites describing the ER as a continuous network of anastomosing tubes running parallel to the longitudinal axis of the dendrite [22] and extends, virtually, into all mature dendritic spines [18],[20]. This hypothesis is further supported by direct recordings from ER in skinned myocytes having an input resistance of ∼850 MΩ and a resting membrane potential around 0 mV between the ER lumen and the cytosol [23] (values of ∼7.5 kΩ/cm^2^ and 15–20 mV were estimated earlier for ER membrane specific resistance [24] and membrane potential [25], respectively). Those studies provide the experimental grounds for suggesting that ER membrane can separate charges and that it exhibits a specific resistance that is similar in magnitude to the plasma membrane (e.g. a typical input resistance for L2/3 pyramidal cells is around 100 MΩ [26] with 20 kΩ/cm^2^ specific resistance for plasma membrane [27]).

In order to test the suggested hypothesis against realistic parameters, provide realistic predictions and enable analytic study of the theory, we developed a mathematical model of a cable-in-cable, thereby generalizing the classical cable theory developed by Rall [28] (Figure 1B and 1C). The model shows that current flow along a system of a cable-in-cable (**CIC**) would, essentially, follow the predictions of the classical cable theory along the *external* cable (i.e. the plasma membrane), but at the same time, would exhibit counter-intuitive properties over the *internal* cable, which cannot be predicted by the classical cable theory. Using the CIC model we show that under realistic parameters the excitatory synaptic activity can give rise to an EPSP-like depolarization across the nuclear envelope, whereas a depolarizing signal initiating at the soma (e.g. action potential) would result in hyperpolarization of the nuclear envelope.

This study provides a novel electrotonic explanation for the ability of the neuronal nucleus to discriminate between orthodromic and antidromic sources of membrane depolarizations. The study further predicts a novel role for compartmentalization of Ca^2+^ within dendritic spines and proposes an additional dimension for synaptic plasticity.

## Results

### The Cable-In-Cable System

The cable-in-cable model principally follows the conventional cable theory and represents the internal membrane system as one passive internal cable that lies within another passive cable of plasma membrane. The key assumptions of the model, ER continuity [17]–[20] and its ability to separate charge similar to the external membrane [23]–[25], rely on reports employing different experimental approaches. To simplify the qualitative description of the CIC theory, the analytical description of the cable-in-cable is reduced into 4 non-dimensional parameters: the ratio between ER diameter and the PM diameter (***E***), the fraction of the non-conductive cross-section (e.g. mitochondria, nucleus) from the PM cross-section (***N***), the ratio between membrane resistivity of ER and PM (***m***) and the ratio between the current *actively* injected into the ER and the current injected into the PM (***I***).

The CIC model can be viewed as an extension to the conventional cable theory, as it collapses to the traditional equations when the internal cable is reduced to zero (*E* = 0) and no axial obstacles are allowed (*N* = 0; for details see ‘Non-dimensional representation’ in the Methods section).

**Figure 2:**
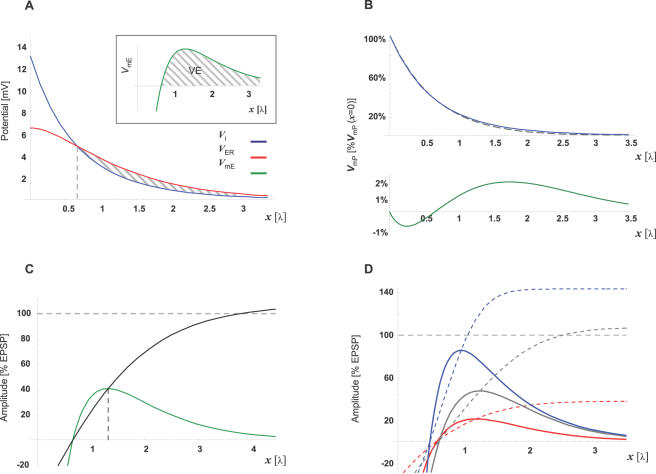
The CIC system predicts a *Virtual-Electrode* along the ER membrane under realistic parameters. Steady-state description of the CIC-model prediction under the parameters described in Table 2. (A) The potential along the cytosol (blue; *V*_i_) and the potential along the ER lumen (red; *V*_ER_) decay at different rates along distance (*x*[λ]) given in electrotonic units. As a result, the ER transmembrane potential (green; *V*_mE_, given by the difference between *V*_ER_ and *V*_i_) generates a qualitatively unique pattern (inset). Namely, *V*_mE_ is negative along ∼0.65 space-constants from the synapse, where it crosses the zero line, becomes positive and then decays to zero *V*_mE_. We name the positive segment (where *V*_mE_>0) that follows a negative segment (where 0<*V*_mE_), ‘*Virtual-Electrode*’ (*VE*; Dashed area, see text for details). Note that practically *V*_mP_ = *V*_i_ (solid red line), since we assume that *V*_e_→0. (B) Comparison between the prediction of the CIC model (solid line) and the prediction of the conventional cable model (dashed line) for the transmembrane potential along the plasma membrane (*V*_mP_). Both traces were normalized to *V*_mP_ at the synapse (*V*_mP(*x* = 0)_). Bottom: Subtracting the prediction of the conventional cable model from the prediction of the CIC model (green line). Note that the maximal difference between the two predictions lies below 2% of *V*_mP(*x* = 0)_. (C) The spatial pattern of the peak potential across the ER membrane (VE-peak) is compared to the EPSP amplitude at the same distance from the synapse (nVE; green) and shows that at the peak, the VE reaches ∼41% of the EPSP. A second way to compare the *V*_mE_ to the EPSP is presenting *V*_mE_ as a fraction of *V*_mP_ at each point along the cable. (black line) This representation of *V*_mE_ shows that at positions beyond the peak of the VE, the *V*_mE_ amplitude reaches values greater than the *V*_mP_ amplitude. (D) Parameter dependency of VE amplitude and pattern: *V*_mE_ was recalculated after increasing (blue) or decreasing (red) *N* or *E* (the non-conductive cross-section or the ER cross-section, respectively) by 15% from the default parameters (gray) provided in Table 2. *V*_mE_ is presented in relation to EPSP as *nVE representation and* as a fraction of *V*_mP_ at each point along the cable (solid lines and dashed lines, respectively).

The CIC system demonstrates a few noteworthy, qualitative properties: (1) The CIC system is governed by two space constants, where both space-constants affect each of the membranes; (2) As the transmembrane potential along the internal cable (***V***_mE_) is given by the difference between two decaying exponents (the potential in the cytosol, ***V*** *_i_*, and the potential inside the ER, ***V*** *_ER_*), it is capable of generating an intriguing non-monotonic pattern as shown in Figure 2A (inset). Namely, localized injection of current into the CIC system would induce depolarization at the external cable and hyperpolarization at the internal cable. However, while in both cases the transmembrane potential would approach zero with distance, the transmembrane potential across the *internal* cable would continue rising with distance, beyond zero, to form a region of depolarization and thereafter it would decay again to zero. We term the area, where a locally-distinct region of depolarization emerges along the internal cable after a segment of hyperpolarization, ‘*virtual electrode*’ (**VE**; Dashed area in Figure 2A; see Discussion for details).

**Table 2:** Parameters, Ranges, and References.

Parameter		Values	Units	Notes and References
*V*_mP[x = 0]_	Amplitude of EPSP (at the synapse)	13	mV	[27]
**Specific parameters**				
R_m_	Membranal Resistance	60	KΩ·cm^2^	[27]
C_m_	Membranal Capacitance	0.8	µF/cm^2^	[27]
R_C_	Cytoplasmic Resistivity	300	Ω·cm	[27]
**Structural parameters**				
d_PM_	Diameter PM	2	µm	
CS_ER_	ER-CS	0.2	PM-CS	0.1–0.2 was estimated [18]
d_ER_	Diameter ER	0.45	d_PM_	*
NCCS	Non-conductive CS	0.33	CS ratio	0.3–0.4 was estimated [18],[22]

**ER**–Endoplasmic reticulum; **PM**–Plasma Membrane; **CS**–Cross-secession.

*: The specific value was calculated to fit the above parameters, CS_ER_ and d_PM_.

### Realistic Parameters Enable a Virtual Electrode over the ER Membrane

Using the realistic parameters described in Table 2, we plotted the steady-state potential inside the cytosolic compartment (***V_i_***) and inside the ER lumen (***V_ER_***) following local current injection into an infinite CIC system. Figure 2A shows that under these parameters the potential along both compartments (i.e. cytosol and ER-lumen) decays gradually towards zero.

Nevertheless, the transmembrane potential along the internal membrane, given by the difference between these two compartments (*V*_mE_≡*V*_ER_−*V_i_*), forms a virtual electrode (inset of Figure 2A). The VE starts and reaches its peak depolarization after a distance of ∼0.6 and ∼1.3 electrotonic units, respectively. The electrotonic unit followed the space constant definition used by the conventional cable theory (defined by Eq. G2.1 in the Methods section), which is equivalent to ∼1 mm using the parameters in Table 2.

The conventional cable theory is well supported by numerous transmembrane recordings. It is therefore interesting to compare the transmembrane potential along the plasma membrane predicted by the CIC model and the transmembrane potential predicted by the conventional model. Figure 2B shows the steady-state change across the *external* cable (plasma membrane; ***V***_mP_) and compares it to the prediction of the conventional cable theory (see ‘Space constant considerations’ in Methods for details). Both models predict exponential decay of transmembrane potential along the plasma membrane, whereas the difference between these two predictions for ***V***_mP_ is negligible (∼1–2%; Figure 2B) and therefore, would be difficult to detect empirically.

**Figure 3:**
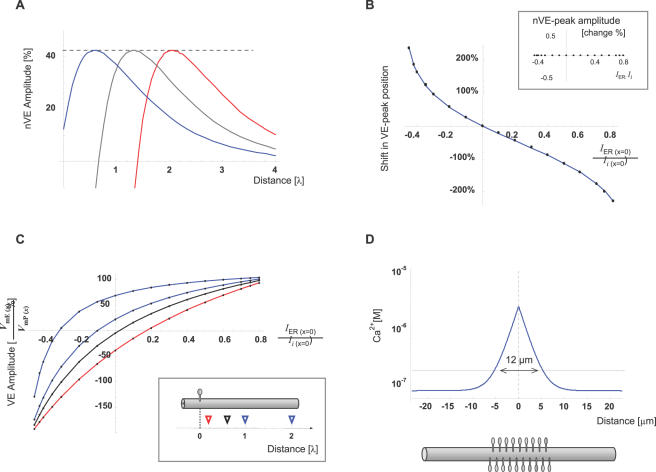
The individual synapse can determine the VE position or its polarity at a distinct target along the cable. (A) Synaptic input initiates, simultaneously, axial currents along the cytosol (*I_i_*) and along the ER lumen (*I_ER_*). The ratio (*I*) between these currents at the synapse (*I_ER_*_(*x = 0*)_ and *I_i_*_(*x = 0*)_, respectively), modulates the position of the VE. These currents may be modified by *active* transition of charges (*i*_mER_; Eq. G1.4, G1.5) across the ER membrane, at the synapse, during synaptic activation (see text for details). The traces represent steady-state nVE introduced by 3 different synaptic signals with identical *V*_mP(x = 0)_ (i.e. potential across plasma membrane at the synapse), identical cable properties, but different *I* ratios (blue, red and black traces represent simulations of synaptic activation with positive, negative and zero *I*, respectively). (B) VE-peak position is presented as a function of *I*. Its position is represented as percent of the distance between the synapse and VE-peak when *I* = 0. Inset: The amplitude of nVE-peak as a function of *I*. Y-axis describes the percent change in the peak level of nVE, from the default (*I* = 0) level. (C) The effect of *I* on *V*_mE_ at several fixed distances from the synapse. Each trace represents the nVE level as a function of *I* at a fixed position (0.2, 0.6, 1, 2 space constants from the synapse; red, black blue and blue, respectively). *V*_mE_ amplitude at each position is described as percent of *V*_mP_ amplitude (EPSP) at that specific distance from the synapse. Inset: Triangles depict the sampling position of traces with corresponding color. Note that at each target, VE amplitude can reach 100% of EPSP level or drop below zero. (D) Steady-state calcium level is plotted as function of distance from a point source of calcium (1 pA). The calculations assumed basal Ca^2+^ level of 70 nM and an endogenous mobile buffer of kD = 50 µM and concentration of 0.5 mM (see text for details). The region with a significant calcium elevation was assumed to be where Ca^2+^ level raised above twice the basal level (dashed line; 12 µm). With a typical dendritic spine density [35],[36] the estimated extent of Ca-signal spread along the dendritic shaft, is predicted to cover a region occupied by ∼20 dendritic spines.

In order to test the significance of the VE amplitude, its peak was compared to the EPSP amplitude. We, therefore, normalized the VE amplitude to the EPSP (*Normalized VE Amplitude*; **nVE**) by dividing ***V***_mE_ by the amplitude of the ***V***_mP_ at the position where the VE-peak occurred (Figure 2C). Thus, the peak amplitude of nVE represents, in percents, the ratio between VE-peak and EPSP at the same position and time. Depending on the specific set of CIC parameters the amplitudes of the VE-peak exhibit EPSP-like levels (nVE range 150%–10%; Figure 2D). Moreover, representing the VE amplitude by nVE is underestimating the relation between VE amplitude and EPSP amplitude, since the ratio between the depolarization across the *internal* cable (the VE amplitude) and the potential along the *external* cable (the EPSP amplitude), gets bigger with distance (Figure 2C and 2D, dashed lines). Thus, the fraction of ***V***_mE_ amplitude relative to the local EPSP amplitude is substantially larger at positions beyond the VE peak. Moreover, when the initial EPSP amplitude (i.e. at the synapse) is altered, the proportion between transmembrane potentials of the internal and external cables is maintained, indicating that the relation between EPSP and VE amplitudes is determined by the specific cable parameters and not affected by changes in synaptic efficacy (see inset for Figure 3B).

An analytic rationale for relating the VE amplitude to ***V***_mP_ arrives from Equations H13.1 and H13.2, which show that at any point in space and time, the amplitudes of both transmembrane potentials are linearly dependent on *V_mP_*_(*x* = 0,*t*)_, the transmembrane potentials across the plasma membrane at the synapse (or dependent on *I_i_*_(*x* = 0,*t*)_, the axial current entering the cytosolic compartment at the synapse; since *V_mP_*_(*x* = 0,*t*)_ = *I_i_*_(*x* = 0,*t*)_·*r_i_*). Namely, the ratio between ***V***_mE **(*x,t*)**_and ***V***_mP **(*x,t*)**_ along a given CIC system (i.e. the pattern over space and time) is fixed and not affected by the magnitude of the synaptic signal.

Thus, introducing a realistic set of parameters to the CIC model predicts that excitatory synaptic activity can give rise to depolarization across the internal membrane, with an EPSP-like amplitude at a realistic distance from the synapse. Moreover, the unique VE-shape of transmembrane potential along the internal cable can explain the ability of the cell's nucleus to differentiate between dendritic origin and somatic origin of a depolarizing signal. Namely, a depolarizing signal that is proximal to the target (e.g. antidromic signal originating from the soma) would *hyperpolarize* the internal membrane at the target (i.e. around the nucleus), whereas depolarizing signals with remote origin would *depolarize* the internal membrane at the target. The effect of the signal along the internal cable (i.e. the VE) would be further subjected to modulations of synaptic efficacy (e.g. LTP or LTD).

### The VE Position Can Be Determined by the Individual Synapse

Evidently, different synaptic inputs originate from a wide range of distances from the cell nucleus. Yet, the VE predicted by the CIC model is essentially a spatial phenomenon that reaches its peak at a fixed distance from the synapse namely, the distance between the VE-peak and the synapse is fixed for any given set of passive cable properties, regardless of the initial potential at the synapse. It is, therefore, interesting to examine its range limits and its significance for distal synapses. For that purpose let us define ***i***_mER_ and ***i***_mPL_, as the currents that are *actively* injected across the ER membrane and across the plasma membrane at the synapse (x = 0), respectively (see Discussion for an actual mechanism which may generate ***i***_mER_ in synchrony with ***i***_mPL_).

As illustrated by the circuit in Figure 1C (and implemented in Eq. D1–D5 formulating the Kirchhoff's law), *I_i_*_(*x = 0*)_ is given by the difference between ***i***_mPL_, the current *actively* entering the cytosol through plasma membrane, and ***i***_mER_, the current *actively* leaving the cytosol into the ER lumen at the synapse; whereas, *I_ER_*_(*x = 0*)_ is given by ***i***_mER_, the current *actively* entering the ER lumen through the ER membrane at the synapse. Along distance, *I_i_*_(*x = 0*)_ leaks out to the external compartment and also into the ER lumen, thereby feeding the axial currents *I_e_* and *I_ER_*, respectively. The rest of *I_i_*_(*x = 0*)_ travels axially along the cytosol as *I_i_* (as illustrated in the inset to Figure 1A and 1C and formulated in the ‘Methods’). Accordingly, ***i***_mER_ represents a supplementary current that is generated by *active* processes at the ER membrane simultaneously with the specific synaptic activity. Positive ***i***_mER_ would, therefore, augment *I_ER_* and diminish *I_i_* whereas negative ***i***_mER_ would diminish *I_ER_* and augment *I_i_*. Alternatively, the effect of ***i***_mER_ may be simplistically illustrated as an increase (for ***i***_mER_>0) or a decrease (for ***i***_mER_<0) of the *V_mE_* at the synapse (*V_mE_*_(*x = 0*)_; red curve in Figure 2A)

The analytical description of the transmembrane potential along the internal cable (Eq. H13.2 in the ‘Methods’ section) suggests that the ratio (***I***) between the initial axial currents at the synapse (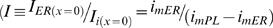; Eq. G1.4, G1.5) can modify the pattern of transmembrane potential along distance. Since this ratio is determined at the synapse, we found it interesting to single out the qualitative features of the VE that are governed by ***I***.

As a first step in answering this question, we used the realistic set of parameters in Table 2, for plotting the effect of ***I*** on the amplitude and location of VE's peak. Figure 3 shows that VE location can be greatly modified by ***I*** (Figure 3A and 3B) with no effect on nVE amplitude (Figure 3B, inset). The ability of the CIC system to generate the VE pattern along the internal cable without active current injection into the ER-lumen indicates that the principle VE pattern can be induced *passively* following synaptic activity, whereas VE location can be further tuned by *active* processes at the synapse-ER complex. Examples for actual processes which may involve negative and positive ***i***_mER_ are activation of Ryanodine receptors and/or activity of the electrogenic SERCA pumps. Both are localized at the ER in the spine heads and both processes can be triggered by excitatory synaptic activity (via calcium influx through activation of glutamate receptors; see Discussion for details).

This ability of an individual synapse-ER complex to determine the VE location may play two roles: First, it may serve as a mechanism for compensating for the wide range of synapse-to-nucleus distances and second, it can introduce a parallel level of synaptic plasticity, which is specifically modulating the synapse-to-nucleus signal in a manner that is largely independent of the efficacy of the specific synapse (i.e. the EPSP). This second level of synaptic plasticity is demonstrated in Figure 3C, where *V*_mE_ level at a wide range of arbitrary distances from the synapse, can be modulated or inversed, solely, by properties of the individual synapse (range: −200% to +100% of EPSP).

Thus, local modulations of the internal compartment at the synapse are capable of introducing a second level of synaptic plasticity, which would modulate the effect of the VE signal on the nucleus. Such modulations could be facilitated by passive properties (e.g. local changes in membrane permeability or surface area of the ER at the synapse) as well as by active properties (e.g. electrogenic pumps and ion channels; see Discussion for details).

### Advantage of a Dendritic Spine-Like Compartment

The majority of the synaptic activity in the cortex is mediated by glutamatergic synapses onto pyramidal neurons. These synapses terminate on mushroom-like structures, dendritic spines. Principally, the above description of VE along the ER can be generated by a synapse located directly on the dendritic shafts. Does the CIC hypothesis predict an advantage of introducing synaptic input via secluded compartments such as dendritic spines?

A plausible answer to this question may be linked to compartmentalization of Ca^2+^ dynamics, which is commonly conceived as one of the main roles of dendritic spines.

Excitatory synaptic activation on a dendritic spine initiates Ca^2+^ influx into the spine mainly through glutamatergic receptors (i.e. N-methyl D-aspartate receptors; NMDAR). Approximately 30% of the Ca^2+^ entering the spine is carried into the ER lumen by the electrogenic [29] Ca^2+^ pump, SERCA (Sarcoplasmic Endoplasmic Reticulum Calcium-ATPase) [11]. Additionally, Ca^2+^ influx into the spine has been suggested to induce Ca^2+^-induced Ca^2+^ release (CICR) from the ER within the spine [30],[31]. Thus, excitatory synaptic activity onto the spine is coupled with positive and/or negative Ca^2+^-mediated currents flowing into the ER lumen at the spine head. In the context of the CIC system, these two processes actively govern the ***I*** ratio presented above.

Under the assumption that VEs play a role in synaptic signaling, one would expect that the synaptic parameter governing their properties (i.e. the ratio ***I***) would be synapse-specific. Namely, different synapses would maintain different ***I*** ratios. In order to achieve this, (1) the ER segment, which directly responds to the increase in cytosolic Ca^2+^, should employ different levels of CICR and SERCA; and (2) the cytosolic Ca^2+^ elevation evoked by synaptic activity should be confined to that specific segment of ER (i.e. confined to the ER segment which binds a particular ***I*** ratio to a specific synapse).

Figure 3D presents the predicted spatial decay of Ca^2+^ level along the distance from a point source of Ca^2+^ in the cytoplasm with endogenous Ca^2+^-buffer. (The endogenous Ca^2+^-buffer parameters were taken from Naraghi *et al.* [32] and the calculations employed conventional models [33],[34]).

This estimate demonstrates that, under realistic spine density of 20–30 spines per 10 µm [35],[36] (up to 60 spines per 10 µm where reported [10] at the distal dendritic branches), activation of a single glutamatergic synapse is expected to trigger calcium-induced currents at multiple surrounding synapses. Namely, in order to enable a synapse-specific ***I*** ratio and comply with the realistic spine density, the spatial expansion of Ca^2+^ elevation should be significantly restricted by at least one order of magnitude.

Therefore, we suggest that compartmentalization of free calcium by the dendritic spines is essential for maintaining synapse-specific tuning of signaling via VE along the internal membrane. This assumption is further supported by experimental evidence indicating that each dendritic spine usually accommodates a single synapse [37],[38].

### VEs Preferentially Converge to the Soma

A pivotal stage in processing synaptic inputs is their convergence and integration at the soma, which leads to initiation of action potentials (APs) and activation of transcription factors at the nucleus. The soma is typically characterized by two anatomical features: (1) it is the widest region of the neuron and (2) it contains the cell nucleus. If VE participates in synapse-to-nucleus signaling it is useful to examine the CIC system at the transition from the dendrite to the soma.

**Figure 4:**
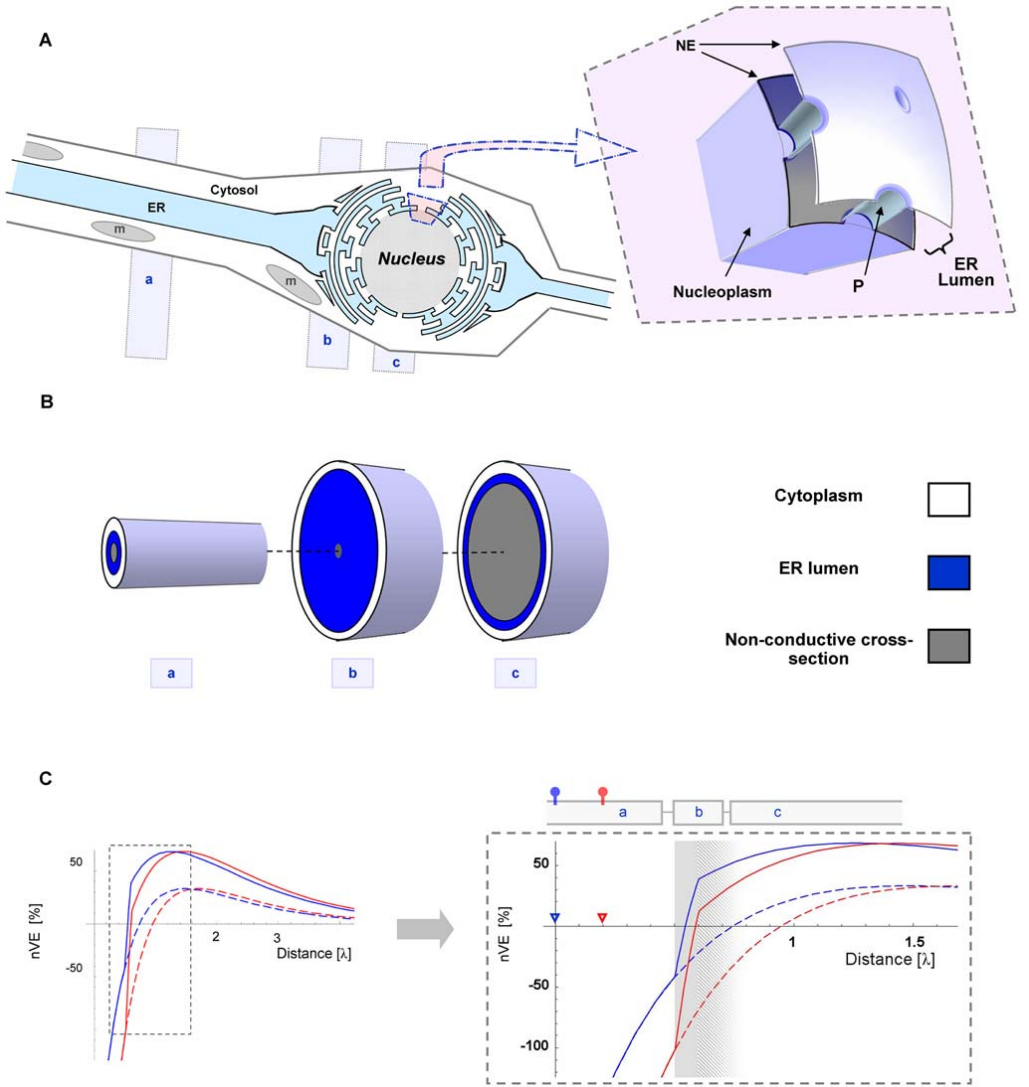
The transition from dendrite to soma compels the VE to converge at the soma. (A) The soma is characterized by a wider diameter and the presence of the cell nucleus. The nucleus is enclosed by two nuclear envelopes (*NE*) and occupies the majority of the cell's cross-section, at its widest diameter. The outer NE is continuous with the ER membrane and the space between the two NE is continuous with the ER lumen (*ER lumen*). The NE allows continuity between the nucleoplasm and the cytosol through pore complexes (*P*), ∼9 nm in diameter. Altogether, the structure of the nucleoplasm and the two nuclear envelopes establishes an electrical continuity with the inner cable. Thus, the electrotonic pathway from the dendritic spine to the cell nucleus may be reduced to three successive CIC systems as indicated by three grey rectangles: (a) dendritic-CIC, (b) perinuclear-CIC and (c) nuclear-CIC. (B) A simplified simulation of the transition from dendrites to soma was conducted by connecting a dendritic-CIC (labeled ‘a’) with somatic-CIC (labeled ‘b’ and ’c’). The dendritic-CIC followed the default parameters (Table 2), whereas the somatic-CIC had a wider diameter (*d*_mP_ = 16 µm) and was further divided into two consecutive segments: perinuclear zone (labeled ‘b’) and nuclear zone (labeled ‘c’). The perinuclear-zone was short (0.2 λ) and characterized by a narrow cytosolic cross-section and wide ER cross-section (*E* = 0.99) and the nuclear-zone was characterized by a wide non-conductive cross-section (*N* = 0.9 and the original *E* value, *E* = 0.45). (C) *V*_mE_ traces of two spatially distinct synaptic sources (red and blue traces) separated by a distance of 0.2 electrotonic units, are plotted with and without the effect of the somatic-CIC (solid and dashed lines, respectively). Each trace is scaled to the EPSP amplitude at the VE-peak (nVE). Right: Vertical expansion into the region of transition from dendritic-CIC to somatic-CIC (shaded zone). The triangles mark the origin (i.e. synaptic source) of each curve by corresponding colors. Note that at the segment that follows the transition from a dendritic-CIC into a somatic-CIC, both traces, which are otherwise negative, become positive and the VE peaks reach higher levels.

In order to model the effect of dendrite-to-soma transition of the VE signal, a second CIC compartment (somatic-CIC) was connected to the CIC system described above (for details regarding multiple CIC systems please find ‘Finite CIC system with arbitrary boundary conditions’ in ‘Methods’). The somatic-CIC construct was aimed at representing the soma at the peri-nuclear region. The peri-nuclear region is characterized by two nuclear envelopes (NE), where the outer envelope is continuous with the ER membrane [39]. These two envelopes enclose the nucleus and form between them a space that is continuous with the ER lumen [39]. The NE allows continuity between the nucleoplasm and the cytosol through nuclear pores (P; about 9 nm in diameter, see Figure 4A). The nuclear pores allow non-selective flux of ions and therefore enable electrical continuity between cytoplasm and nucleoplasm. For illustrating the VE at the peri-nuclear region the somatic-CIC had wider diameter (16 µm) and included an initial part with narrow cytosolic cross-section (representing the perinuclear area) followed by a part with larger non-conductive cross-secession, representing the nucleus (Figure 4B). Except for these parameters the others parameters were kept as described above (Table 2).

Figure 4C shows a simulation of two VE signals arriving at the soma from two electrotonically-dispersed synapses. It illustrates (in Figure 4C, right) that the transition from a dendritic-CIC into somatic-CIC may act on disperse VEs as a “converging lens”. Namely, amplifying the VEs amplitude and converging them to the soma. This converging effect of the soma preserves the ability of current ratio at the dendritic spine (***I***) to modulate the VE amplitude and actual manifestation at the nucleus.

### Time Domain Aspects

The VE pattern displayed above represents the steady-state difference between potentials in the ER-lumen and in the cytosol. However, since actual synaptic currents are confined in time, the validity of a steady-state description of VE needs to be evaluated at the time scale of synaptic input duration.

**Figure 5:**
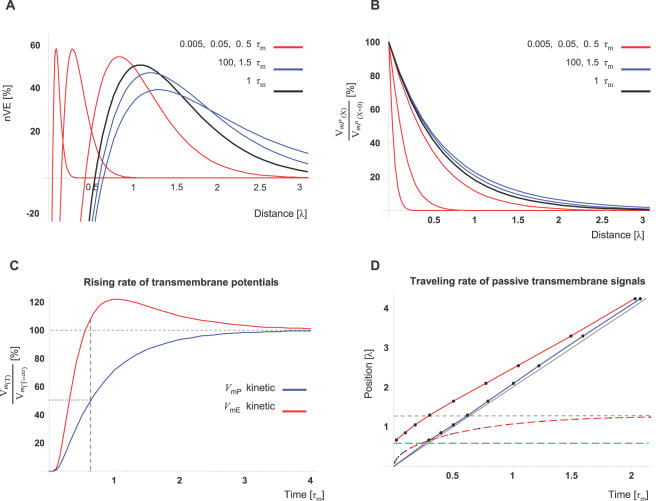
Time domain analysis of CIC system. (A) *V*_mE_ traces at different time points after starting to depolarize the synapse (*V*_mP[x = 0]_ = 13 mV). Note that the VE pattern is already established at *T* = 0.005 (units of time-constant; τ_m_ = 48 ms) and that the VE-peak virtually reaches its final position and amplitude within 1.5 τ_m_ (72 ms). Each of the *V*_mE_ traces is presented as percentage of the EPSP level at the position of the VE-peak (nVE), at the specific time point. The black trace depicts the *V*_mE_ at time points of *T* = 1 whereas red and blue traces depict the *V*_mE_ at time points lower or higher than *T* = 1, respectively. (B) For comparison, the conventional pattern of an EPSP along distance (*V*_mP(X)_) is plotted for the same time points as in A. The amplitude of each of the *V*_mP_ traces is expressed as percentage of the potential at the signal's origin (synapse; *X* = 0). Color representation of the different time points is similar to (A). (C) The rising rate of *V*_mE_ or *V*_mP_ to steady-state level (red or blue, respectively) is simulated at the position of the VE-peak (*X* = 1.29). The amplitude is expressed as percent of the steady-state level at that position. Note that at the time *V*_mP_ reaches 50% of its steady-state level, *V*_mE_ has already reached its steady-state level and starts overshooting this after 0.6 τ_m_ (29 ms) *V*_mE_ reaching peak of ∼20% above the steady-state levels at 1 τ_m_ (48 ms). Note that these kinetics lies within the duration of a synaptic current influx induced by a typical glutamatergic synapse (12–24 ms; see text for details). (D) The propagation pattern and rate of electrotonic signals along the internal cable (red; *V*_mE_) and along the external cable (blue; *V*_mP_) compared to the prediction of the conventional cable theory (gray; *T* = 2⋅λ). Following the conventional definition for electrotonic velocity we plotted, over time, the points where the transmembrane potentials reached 50% of its steady-state level (solid lines). Dashed red line depicts the rate the VE-peak approaches its steady-state position (dashed, horizontal gray line). At different positions along the internal cable, the potential develops toward positive or toward negative directions (as demonstrated in Figure 5A). For simplicity, only the region where *V*_mE_ develops toward a positive direction was analyzed. This region lies above the dashed green line, which depicts the position where *V*_mE_ = 0 at steady-state (*X* = 0.64 λ). Note the negligible difference between the predictions of the CIC model and the conventional model for the speed of electrotonic signals along the external cable (blue and gray lines, respectively).

To address this question, we simulated the ER-membrane potential at several time points after the beginning of synaptic activity. We described CIC dynamics by units of the conventional membrane time constant (**τ**_m_; τ*_m_*≡*R_m_C_m_*), which is equivalent to 48 ms under our specific parameters (Table 2). Indeed, Figure 5A shows that VE pattern is not a unique steady-state phenomenon, as it is already established within 0.005 time constants (equivalent to 0.2.5 ms) whereas the amplitude of its peak develops over time similarly to the development of an EPSP over time (Figure 5B). Figure 3A(dashed line) shows that while the VE travels (electrochemically) along the internal cable, the ratio between the amplitudes of the VE-peak and EPSP (nVE) is higher than the steady-state ratio at the final position of the VE-peak.

In order to get a better estimate of the VE kinetics over time, we compared (Figure 5C) the change over time of the two transmembrane potentials (*V*_mE_ and *V*_mP_) at the position of the VE peak (*X* = 1.29; the peak of the blue trace, *T* = 100, in Figure 5A). We therefore used the conventional cable theory [40] for simulating the development of plasma-membrane potential, *V*_mP_, over-time (as described explicitly in Eq. I1 in the Methods).

Using the conventional spatio-temporal solution described by Jack *et al.* [40] (Eq. I1) and the conventional definition for traveling speed of electrotonic signals, Figure 5D, reveals that *V*_mE_ amplitude rises, to its steady-state level, slightly faster than EPSP amplitude. Moreover, despite the fact that the electrotonic time constants are the same for both the inner and outer cables, the VE pattern and its amplitude are established dramatically faster than the EPSP, at the segment around VE-peak (Figure 5C and 5D). The fact that at the position of VE-peak, *V*_mE_ amplitude reaches its steady-state level within ∼0.6 time constants (29 ms) and thereafter overshoots its steady-state peak by 20% shows higher efficacy for VE as electrotonic signal for signal durations around 1 time constant. This is in line with the typical duration of the synaptic current induced by glutamatergic synapse (EPSC's time to 50% decay: ∼10 ms [41]) and the expected time for it to spread out to that position.

In conclusion, time domain analysis shows that electrotonic signaling by means of VE along the internal cable has kinetics that are similar and slightly faster than electrotonic kinetics of the EPSP along the external cable. The comparable kinetics indicates that VE can, similarly, convey synaptic signals induced by realistic synaptic current duration. Altogether, time domain analysis demonstrates that the steady-state analysis provides plausible representation of VE.

## Discussion

This biophysical study provides a new explanation for the remarkable ability of the pyramidal cell nucleus to differentiate between orthodromic depolarizing signals and antidromic depolarizing signals [1],[6]. We show that depolarization of the cell membrane is accompanied by two opposite and position-dependent effects on the internal membrane: (1) passive hyperpolarization of internal membranes at the region where cell depolarization initiates, and (2) passive depolarization of the internal membrane at some distance from the point where depolarization initiates. This implies that a depolarization of cell membrane, by means of current influx at the soma, is predicted to induce hyperpolarization of the ER at the soma (namely, the nuclear envelope); whereas a distal current influx (i.e. at the dendrites) would induce depolarizing effect on the nuclear envelope (in the form of a VE). We further show that the distance between the origin of the depolarization (synapse) and the position where VE appears, can be modified at the single spine head, by passive properties (such as the surface area of the spine apparatus) and by active properties (such as the Ca^2+^-dependent current influx through SERCA pumps or CICR through the ryanodine and IP3 receptors). Finally, by feeding our simulation with realistic parameters (from the literature), we show that the amplitude, the time-constants and the space constants of the VE exhibits scales similar to that of an EPSP.

Taken together, these electrotonic considerations introduce a qualitatively new mechanism of intracellular signaling operating at electrotonic time scales of EPSP [41].

### Dendritic ER Architecture Supports Axial Conductance

ER is conventionally regarded as an unstructured network of tubes and sacs. Thus, an equivalent cylinder with a cross-section similar to the axial cross-section of the ER network may misrepresent the effective axial resistance along the ER lumen. For example, a cross-section measurement of a *single* tube tangled in a bigger volume will appear misleadingly higher than the actual cross-section available for an axial current traveling along that tube, generating an underestimation of the actual axial resistance along that tube. Thus, lumen cross-section in an unstructured network can not faithfully represent the effective axial cross-section.

However, a detailed structural study of the neuronal ER architecture by Martone *et al.* [22], reveals that the ER in the dendrites of a spiny neuron forms a network of tubules running in parallel to the longitudinal axis of the dendrite. Thus, dendritic ER architecture appears to support axial conductance, whereby axial ER cross-section provides a more realistic estimation for its axial resistance.

### VE-Mediated Signal Transduction to the Nucleus

A key question that lies beyond the focus of our electrotonic model is: how would a depolarization at the ER pass a signal into the cell nucleus?

One plausible route may be electrotonic signals across the nuclear envelopes (**NEs**). Since the ER lumen is continuous with the lumen between the inner and outer nuclear envelopes and the nucleoplasm is continuous with the cytosolic compartment via holes (i.e. the nuclear pores) through the NEs, the transmembrane potential across the NEs follows the transmembrane potential changes across the ER (namely, VE). Thus, voltage-sensitive properties across the ER membrane forming the outer nuclear envelope, may mediate the signal into the nucleus. This proposal for nuclear signaling is in line with reports about several types of voltage sensitive ER channels [23], [42]–[46] provide partial support this possibility.

A second option is an initiation of locally-distinct perinuclear, Ca^2+^ signals, which may have a bearing on nuclear moieties. A single VE or sequence of multiple VEs may initiate and modulate theses signals through activation of voltage-sensitive properties cross the ER membrane. The fact that Ca^2+^-elevation in the nucleus is necessary for numerous nuclear activities and specifically activity-dependent CREB phosphorylation [3],[47], implies that voltage-activated Ca^2+^-channels may initiate a local Ca^2+^ signal that will be amplified and modulated by Ca^2+^-activated Ca^2+^-channels. This possibility is supported by (1) experimental reports from the CNS [43] and from non-nerve tissue [44] describing ER Ca^2+^ channels which increase their opening probability sharply by depolarization; and (2) studies showing that the inner NE expresses intracellular Ca-activated Ca release channel, i.e. inositol 1,4,5-trisphosphate receptors (IP_3_Rs) and ryanodine receptors (RyRs) [48],[49].

Moreover, this explanation is consistent with various experimental observations showing that: (1) locally-distinct cytoplasmic events of ER-Ca^2+^-release (e.g. Ca^2+^ puffs), originating within a 2–3 micron perinuclear zone, appear to initiate Ca^2+^ elevation in the nucleus, [50] (2) The NE is a functional calcium store [51],[52] and Ca^2+^ signals within the nucleus can be evoked in the absence of elevation in cytosolic Ca^2+^ [48],[51],[52].

Thus, co-localization of VE with voltage-sensitive channels [23],[42],[45],[46], voltage-sensitive calcium channels and calcium-sensitive calcium channels [48],[49] along the ER at the nuclear envelopes, is one possibility for instantly coupling VE with perinuclear and nuclear calcium signals. Such machinery introduces a new layer of Ca^2+^-mediated control of nuclear function in neurons and, possibly, in non-neuronal cells.

### Synaptic Plasticity of Spine-to-Nucleus Signaling

Modification of the effect that a specific synaptic activity has on the postsynaptic cell is conventionally termed synaptic plasticity. This theoretical study shows that a synapse-specific property, the ***I*** ratio (defined in Eq. G1.4), can modulate the effect a specific synaptic activity has on the transmembrane potential across the nuclear envelopes of the postsynaptic cell. Modification of the synapse-specific ***I*** ratio may, therefore, represent a second level of synaptic plasticity. Figure 3C shows that regardless of the strength of the signal across the plasma membrane (e.g. EPSP), the signal across the internal membrane (VE) at an arbitrary distance from the synapse can be set exclusively by the ***I*** ratio to be positive, negative or zero. Nevertheless, the *magnitude* the VE signal will be subjected to the conventional synaptic plasticity as well (i.e. potentiation or depression of EPSP). This suggests that the synapse-to-nucleus signal bares the capability for independent synaptic plasticity at various ranges of electrotonic distances between synapse and nucleus.

One plausible physiological mechanism, which may sustain a synapse-specific *I* ratio modulation, may be electrogenic calcium fluxes across the ER membrane at the synapse. This suggestion is inline with the fact that a typical EPSP in the cortex, which is generated by glutamatergic synaptic activity onto dendritic spines, involves calcium influx from the extracellular compartment into the specific spine head. The extension of the ER into the spine head (spine apparatus) exhibits capabilities to link elevation in spine-calcium into inward or outward currents across the ER at the spine. Inward calcium-dependent current across the ER may be mediated by the electrogenic activity of SERCA pumps [29], whereas outward currents across the ER in the spine head may be mediated by Ca^2+^-sensitive channels [31]. Immunocytochmical studies shows that RyR labeling is notable in dendritic spines of cortical pyramidal cells, whereas their dendritic shafts are mostly unlabeled [53].

A large body of theoretical studies supported by experimental data [11], [54]–[57] shows that the interaction between SERCA, RyR, IP3R, endogenic buffers and intracellular calcium stores can generate a wide variety of Ca^2+^ dynamics, which are fundamentally dependent on the temporal pattern of Ca^2+^-inputs. Taken together, the spine head seems to contain the hardware necessary for generating synapse-specific modulation in VE, which may be further modified by the pattern of the specific synaptic input. This assumption may be further supported by the facts that: (1) the majority of excitatory communication in the cortex is mediated via dendritic spines which are structures that can compartmentalized Ca^2+^ [11]; (2) each spine head receives a single glutamatergic synaptic input [37],[38]; and that (3) pyramidal neurons, the main source for glutamatergic synaptic inputs in the cortex, respond to their preferred sensory input by burst patterns of action potentials.

Thus, the CIC prediction for the *I* ratio combined with the current knowledge on excitatory synaptic signaling in the cortex, provide circumstantial support to the existence of synaptic plasticity of the spine-to-nucleus signaling, which may be further modified by the pattern of the specific synaptic input.

### Model Predictions Are Stable over a Wide Range of Parameters

**Figure 6:**
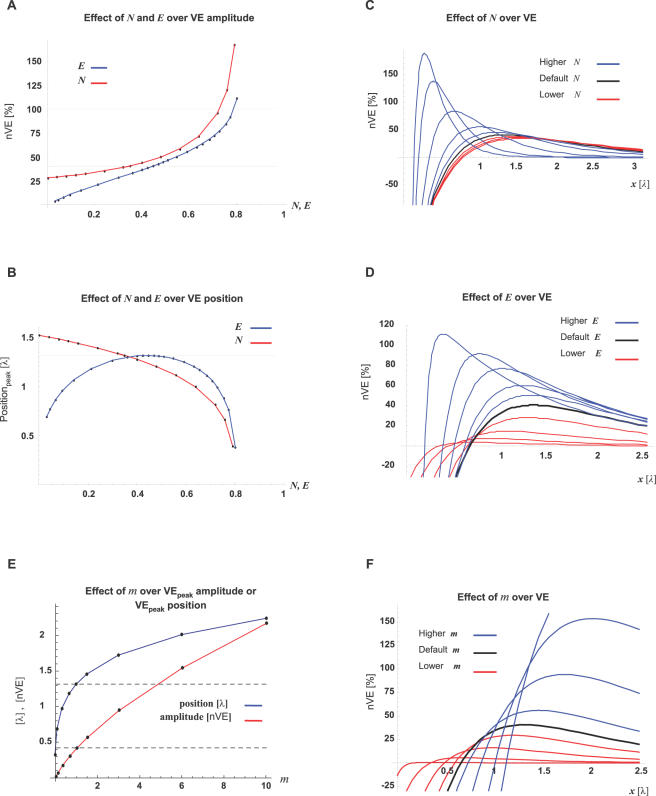
VE can be generated by a wide range of parameters. One-dimensional mapping (i.e. one parameter at a time) of the fraction of the non-conductive cross-section (*N*) and the fraction of the ER cross-section of the total cross-section (*E*). All other parameters followed the default parameters (Table 2). VE amplitude is presented as percent of the EPSP amplitude at the position of the VE peak (nVE). Note that *E* and *N* describe each a fraction of the total cross-section, therefore their range is limited by: {*N*+*E*≤1}. (A) Levels of nVE-peaks are presented against different *N* (red trace) or *E* (blue trace) values. (B) The positions of the VE-peak are plotted against different *N* (red trace) or *E* (blue trace). (C,D) VE patterns are sampled across the range of *E* and *N*, tested. Traces generated with parameters above or below the default values are presented in blue or red, respectively. (E) Amplitude and position of the VE-peaks are presented against different *m* values. The red trace represents amplitude (nVE units). The Blue trace represents the position (λ units). Gray dashed lines mark the amplitude and position of the default value (*m* = 1). (F) VE patterns are sampled across the range of *m*, tested. Traces generated with *m* above or below the default value are presented in blue or red, respectively.

Evidently, the VE pattern and amplitude is parameter-dependent. For ruling out the possibility that the model's predictions are specific to a narrow range of parameters (as described in Table 2), we evaluated the robustness of its predictions over a wide range of parameters. Using one-dimensional parameter-mapping, we show (Figure 6) that VE along the internal cable is a reliable phenomenon and its amplitude has an EPSP-like magnitude.

For simplicity, we focused our study on analytical description of the CIC theory and therefore we have neglected the role of ER curvatures. Likewise, for simplifying the time-domain analysis, we have assumed that the inner and outer the cables have identical time-constants (**τ_m_**). Nevertheless, we allowed different membrane-*specific* resistivities for ER and PM (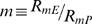; Eq. G1.2), and kept the similar time constant for both PM and ER membranes by constraining the relation of the two membrane-*specific* capacitances (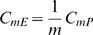; Eq. H8). This constraint over the relation between the two membrane-*specific* capacitances does not affect the analytical analysis of different membrane resistivities at steady-state.

One of the major features of the ER, which was neglected in our study, is the network structure of the ER. One may ask whether this simplification can breach the prediction of the CIC model?

Apparently, the principal prediction of the CIC model, the VE, is in line with a model specifically developed for describing a network of passive cable elements, [58] the unequal anisotropic bidomain model (for review see [59]). Moreover, virtual electrodes predicted by the bidomain model have been demonstrated empirically over cardiac myocardium [60],[61]. Thus, the ability of a network of passive cable elements to generate VE, is well supported.

### Some CIC-Model Predictions

The CIC theory provides several experimentally testable predictions. Interestingly, we found that each prediction can be supported (at least partially) by recent experimental reports.

The first prediction resolves the question presented above, regarding the traveling speed of the ‘synapse-to-CREB’ signal. The CIC model predicts that (1^st^ prediction) synapse-to-nucleus signaling would exhibit an electrotonically-fast propagation velocity that is 2 or 3 orders of magnitudes higher than expected from a regenerative Ca^2+^-wave or diffusion of a second messenger (i.e. kinase-bound CaM, proposed previously), respectively (see Table 1). This prediction is in line with the ‘synapse-to-CREB’ time (∼15 seconds) reported by Mermelstein *et al.* [8]. Moreover, this prediction expands our ability to comprehend the way synapses, as myriad sources of fast electrical signals, communicate their information stream to the distant nucleus.

The CIC model suggests that active properties within the spine heads (e.g. Ca^2+^-mediated currents across the spine apparatus, via SERCA pumps, ryanodine receptors and IP3 receptors) encode an additional level of synaptic plasticity by determining the efficacy of the VE at the nucleus. This suggests that (2^nd^ prediction) as an information-encoding parameter, spine Ca^2+^-dynamics would exhibit high variability between spines in the same cell and spines in the same cell group. Namely, measurements of the fraction of Ca^2+^, which enters the ER at the spine head following excitatory synaptic activity, would show a wide range of values between spines of similar neurons at similar location. This prediction is supported by Sabatini *et al.* [11] who measured the fraction of Ca^2+^ entering the ER at the spine head of CA1 pyramidal neurons.

The CIC model shows that the ability of a dendritic spine to induce effective VE at the nucleus is impaired if the spine is too close to the cell nucleus. Therefore, some properties of dendritic spines are predicted to undergo gradual change in relation to their distance from the nucleus. For example (3^rd^ prediction) spines, which otherwise exhibit high density along the dendrites, should be absent from the soma and proximal part of the dendrites, which has been observed in several studies [9],[10],[35],[37],[62],[63]. Likewise, it is expected (4^th^ prediction) that on average, a proximal spine would exhibit a lower activity of CICR and/or higher activity of SERCA, compared to a dendritic spine located at remote dendritic regions. Similarly, for facilitating the passive conductance of synaptic currents into the ER, (5^th^ prediction) the ER branch at the spine head (the spine apparatus) should exhibit varying degrees of laminar organization and increased surface area compared to the spine head enclosing it [64]. For example, the ratio between the surface area of the spine apparatus and spine head would be (on average) greater for spines which are distal from the nucleus. These last two predictions should be testable by appropriate physiological, immunocytochemical and morphological experiments.

Finally, while activation of glutamatergic synapses at the dendrite induces robust CREB phosphorylation at the nucleus, (6^th^ prediction) a concomitant activation of extra-synaptic glutamatergic receptors at the soma would suppress the electrotonic induction of VE at the nucleus and therefore suppress CREB activation. This prediction is supported by Hardingham *et al.* [65] who showed that, while synaptic activation of glutamatergic synapses induces CREB phosphorylation, bath application of glutamate suppresses it.

One way of obtaining direct experimental evidence is to apply the patch clamp technique for recording and manipulating the transmembrane potential simultaneously across both the ER and the PL, during synaptic activity. This can be achieved by employing the pipette-within-pipette patching technique described by Jonas *et al* [66]. Although this approach would be technically challenging, its successful application would enable a simultaneous recording of the two transmembrane potentials. Such an experiment would directly address the question of whether a direct interaction is present or not.

In summary, the significant contribution of the current study is proposing a VE along the ER membrane as a means of ultra-fast intracellular signal transduction and demonstrating its feasibility under realistic parameters using a cable-in-cable model. The CIC hypothesis presented here contributes also by introducing the possibility of an additional level of synaptic plasticity and a new perspective for the role of dendritic spines, which densely populates the dendrites of spiny neurons. Since ER is continuous also in non-neuronal cells, electrotonic signaling along internal membranes may act as a general means of fast signaling between cell periphery and nucleus and other sub-cellular compartments.

This study shows that intracellular level biophysical theory may introduce concepts and principles that appear counter-intuitive with views originating from conventional cellular level electrophysiology, suggesting that the phenomenological richness of intracellular architecture and the associated electrophysiology may still offer surprises.

## Methods

### Model Assumptions

The model follows the classic cable theory [40],[67],[68] and introduces a model of a cable in cable. We used Mathematica5 (Wolfram Research) for numerical calculation and for checking the analytical derivations.

Model assumptions are:

1. The ER network can be reduced to a single passive cable that goes through the main dendritic shaft. (i.e. Cable-In-Cable; CIC)
2. Both cables are perfect cylinders and parameters are assumed to be passive and uniform throughout.
3. The model follows the three compartmental circuits described in Figure 1C.
4. The model represents membranes around their resting potentials. Therefore, trans-membranal ionic currents are approximated to be passive, governed by membrane resistance and capacitance (r_m_ [Ω•cm] and c_m_ [F/cm], respectively).
5. Data obtained from skinned muscles suggests that ER can separate charge with resistivity that is comparable to the plasma membrane (PM) [23].
6. EM reconstruction of neuronal dendrites shows that the dendrite is occupied by structures that may partially obstruct axial conductance (e.g. mitochondria and transport vesicles; Figure 1B). Therefore, the model assume that the conductive cross-sections of the dendritic cytosol is smaller than the anatomical cross section. (see Table 2)
7. The effect of synaptic activity was assumed to initiate at the point were spine neck connects to the dendritic shaft, and was assumed simultaneous for both Cytosol and ER lumen.
8. Sign conventions for currents: (a) Outward trans-membranal current is positive (for both membranes). (b) Positive injected current drives *V*_m_ in a positive direction. (c) Axial current flow periphery-to-center is positive. (i.e. Lt-to-Rt in the schema).

### Cable-In-Cable Model

The equations below follow the circuit in Figure 1C. The parameters and their definitions are provided in Table 2.

A. Ohmic Axial current:
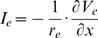
(A1)

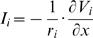
(A2)

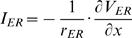
(A3)


B. Total axial current (*I_T_* ) is constant:
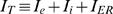
(B1)


C. Radial (trans-membranal) currents in a cable with no additional current source:
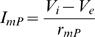
(C1)

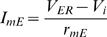
(C2)


D. Kirchhoff's law: (Inward current is defined, negative)
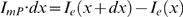
(D1)

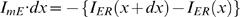
(D2)


Therefore:
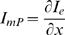
(D3)

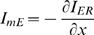
(D4)

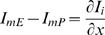
(D5)A system of ODE is obtained from combining all the above:
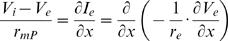
(E1)

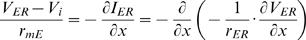
(E2)

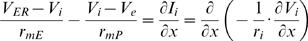
(E3)The system can be represented as: 
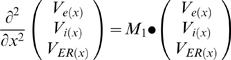
where
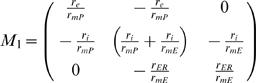
(E4)The general steady-state solution is characterized by two space constants (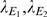) given as the sum of two decaying exponents:
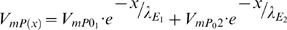
(F1)

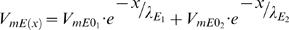
(F2)For the explicit solution, see ‘*Time Domain*’ below.

### Non-Dimensional Representation

We found it advantageous to describe the solution (Eq. F1 and Eq. F2) by four non-dimensional and independent parameters. For that purpose we defined the following parameters (Eq. G1.1–G1.4):

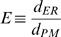
, represents the ratio between ER and PM diameters; {0≤*E*<1}

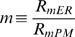
, represents the ratio between the *specific* resistivities [Ω·cm^2^] of ER and PM membranes; {0<*m*}

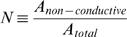
, represents the ratio between of the non-conductive cross section and the total cross section; {0≤*N*<1}

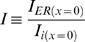
, represents the ratio between currents *actively* injected into the ER lumen and cytosol at the synapse {*x* = 0}

Note that 
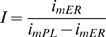
 (Eq. G1.5; see text for details)

1. where
2. *i_mPL_* is the current *actively* crossing the PL into the cytosol, at the synapse{*x* = 0},
3. *i_mER_* is the current *actively* crossing the ER from the cytosol into the ER lumen at, the synapse{*x* = 0}.

The conventional cable theory defines the space constant (λ) as: 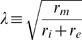. Under the assumption that *r_e_*→0, λ is often represented as 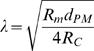. In order to avoid non-specific parameters (*r_i_*,*r_m_*) we followed the second representation and defined:
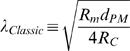
(G2.1)Non-dimensional scaling is obtained by defining:
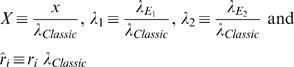
(G2.2 - G2.5)


### Time Domain

For non-steady state conditions the trans-membranal current of cylindrical cable includes transient capacitance currents and is given by:
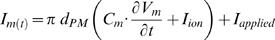
(H1)where:

1. *I*_m_ is the trans-membranal current per length (*d*x). [A/cm]
2. *I*_ion_ is the Ohmic ionic current per membrane surface. [A/cm^2^]
3. *I*_applied_ is the current applied by external sources. [A/cm]

Accordingly, *I*_mP_ and *I*_mE_ can be described as
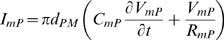
(H2)

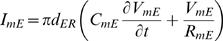
(H3)The ODE system becomes
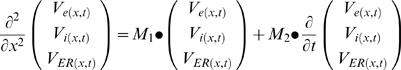
where:

*M*_1_ is as described above (Eq. E4)
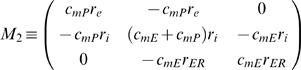
(H4)For enabling the analytical solution we assumed similar time constant (τ*_m_*) for both PM and ER membranes. We, therefore, defined *specific* membrane parameters (*C_m_*,*R_m_*): *τ_m_*≡*R_m_*·*C_m_*, *C_m_*≡*C_mP_* and *R_m_*≡*R_mP_* (Eq. H5–H7) (Units: sec, F/cm^2^, Ω·cm^2^, respectively).

We allowed different membrane-*specific* resistivities for ER and PM (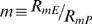; Eq. G1.2), and forced a similar time constant *R_mE_*· *C_mE_* = *τ_m_* ( = *R_mP_*· *C_mP_*) for both PM and ER membranes by assuming:
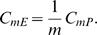
(H8)This assumption (Eq. H8), which is taken for enabling an analytical solution for the time-domain (see below), do not affect the steady-state solution.

By incorporating the *specific* membrane parameters (Eq. H5–H8), matrices M_1_ and M_2_ become:
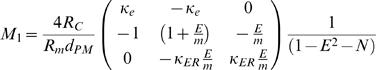
(H9)

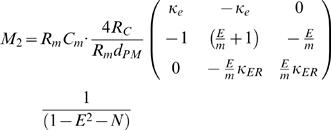
(H10)


where:
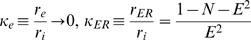



The system becomes:
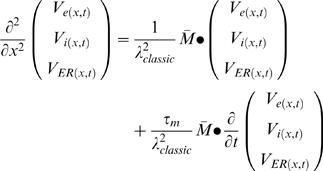



where:
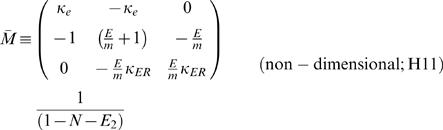
The non-dimensional representation of the system is:
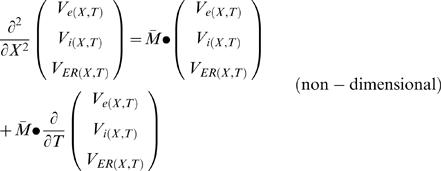



where:
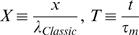



Eigenvalues of 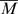:
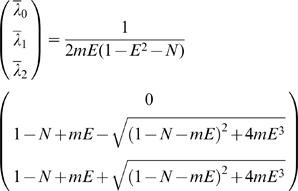
(H12)where: κ_e_→0

The explicit solution (obtained analytically using Laplace transform) [40] is:
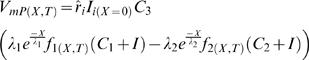
(H13.1)

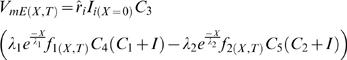
(H13.2)where *V_mP_*_(*X*,*T*)_≡*V_i_*_(*X,T*)_−*V_e_*_(*X*,*T*)_ and *V_mE_*_(*X*,*T*)_≡*V_ER_*_(*X,T*)_−*V_i_*_(*X*,*T*)_. (Note that *V_mP_*_(*X*,*T*)_ = *V_i_*_(*X*,*T*)_ under the assumption that *V_e_*_(*X*,*T*)_→0)

where ƒ is a non-dimensional function of *X* and *T*:
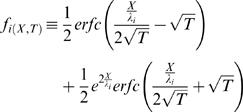
(H14.1)


where *erfc* is the complementary error-function: *erfc*(*x*)≡1−*erf*(*x*)

1. *erf* is the error-function.
2. *i* index (values: 1 or 2)

Note that at steady-stat (*T*→∞) : *f_i_*_(*X*,*T*)_→1

where **λ_1_**, **λ_2_** are non-dimensional space constants:
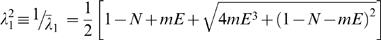
(H14.2)

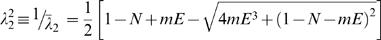
(H14.3)


**Note** that for any realistic set of parameters: {0≤*N*<1,0≤*E*<1, 0<*m*, 0<(1−*N*−*E* ^2^)}

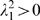
 and 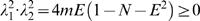, and therefore: 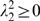.
- ((1−*N*−*mE*)^2^+4*mE* ^3^)>0 and therefore: 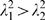.

As a result: λ_1_ and λ_2_ has real solution

where *C*_1_–*C*_5_ are constants defined as followed:
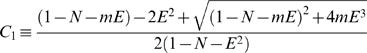
(H14.4)

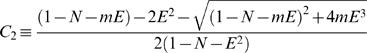
(H14.5)


(Note that (1−*N*−*E* ^2^) describes the fraction of the cytosolic cross-section and therefore: (1−*N*−*E* ^2^)>0)
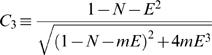
(H14.6)

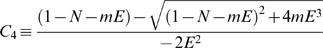
(H14.7)

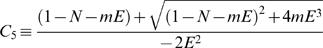
(H14.8)
**Note** that when the internal cable collapses to zero (***E*** = 0) and no axial obstacles are allowed (***N*** = 0), the system collapses to the conventional cable equation.

Namely, 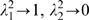, *C*_1_→1, *C*_2_→0, *C*_3_→1, (*C*_4_,*C*_5_ are not defined) and make Eq. H13.1 collapses into the traditional solution: 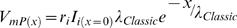. It can be shown that when ***E*** = 0, the CIC system collapses to the conventional cable equation, for any realistic ***N***: {0≤*N*<1} (see ‘Space constant considerations’ below, for details).

Where the electrotonic kinetics predicted by CIC model are compared with those predicted by the conventional cable theory, we followed the conventional spatio-temporal solution described by Jack *et al.* [40]:
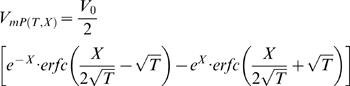
(11)


where *erfc* is the complementary error-function: *erfc*(*x*)≡1−*erf*(*x*).

1. *erf* is the error-function.
2. *V_0_* is the steady-state membrane potential at the synapse.
3. *X* is distance in non dimensional units 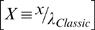, as described in Eq. G2.1, G2.2.
4. *T* is time in non dimensional units 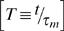, as described in Eq. H5.

### Space Constant Considerations

The classic cable theory assumes no obstacles for the axial current. It practically defines an *effective* axial intracellular resistivity, *R_i_*, which is already adjusted (empirically) to the actual non-conductive cross-sections (e.g. mitochondria) along the specific cable. In contrast, the CIC model incorporates an independent, axial non-conductive cross-section. Therefore, the CIC model assumes that the axial intracellular resistivity represents a cytoplasm without non-conductive cross-sections. Evidently, this deviation from the convention is inevitable if axial obstacles should not be omitted from the CIC model. This difference in terminology can be rectified as described blow.

The relation between *R_i_*, *R_C_* and non-conductive cross-sections (***N***) along the cable becomes:
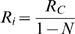
where *R_C_* is the axial intracellular resistivity, specific for a cytoplasm without non-conductive cross-sections.

1. *R_i_* is the *effective* axial intracellular resistivity (adjusted to the actual non-conductive cross-sections along the specific cable)
2. *N* is the fraction of the non-conductive cross section from the total cross-section.

Accordingly, *N* is incorporated in the conventional space-constant (λ*_N_*) as:
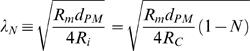
Thus, when the internal cable collapses to zero (*E* = 0) *and* axial obstacles are allowed {0≤*N*<1}, the system collapses to the conventional cable equation with space-constant *λ_N_*.

Namely, 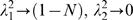, *C*_1_→1, *C*_2_→0, *C*_3_→1, {*C*_4_,*C*_5_ are not defined} which makes Eq. H13.1 collapses into the traditional solution [40], formulated in Eq. I1:
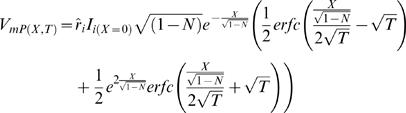
or
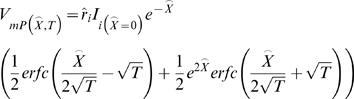
where 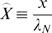.

In Figure 2B we compared the potential along the external cable (*V*_mP(X)_) predicted by the classic theory and the *V*_mP(X)_ predicted by the CIC model. In that calculation we followed an empirical definition and defined the classic-model's space constant by fitting a single exponent to two points along the CIC prediction for *V*_mP(X)_. The first point was X = 0 (*V*_mP(X)_ =  *V*_mP(0)_) and the second point was arbitrarily chosen as the point where *V*_mE(X)_ = 0. Nevertheless, we also tested a second approach for defining a single space constant to the CIC system using *λ_N_* (as described above). Under both approaches the difference between the two predictions is too small to be detected experimentally (within range of few percentages of the initial potential, *V*_mP(0)_ ).

### Finite CIC System with Arbitrary Boundary Conditions

Explicit solution for finite CIC at steady-state with arbitrary boundary conditions.

Boundary conditions: *V_mP_*_(0)_, *V_mER_*_(0)_ Initial potentials at: *X* = *0*

 *V_mP_*_(*L*)_, *V_mER_*_(*L*)_ Ending potentials at: *X* = *L*

The explicit solution:
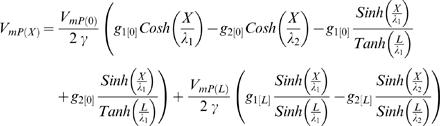


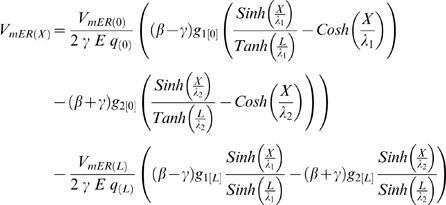
where 
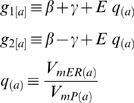



*a* is an index that takes values *0* or *L* in the expressions above
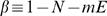


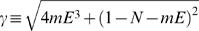
When {*L*→∞}, the above explicit solution for finite CIC gives the CIC solution for semi-finite CIC (provided in Eq. H13.1–H14.8) at steady state:

where 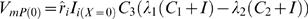 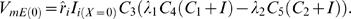


### CIC's Input Resistance

Within the classic cable-theory the conventional definition for input resistance (namely the ratio between potential and current at the point where *X* = *0*) provides a constant parameter, which is solely determined by structural cable properties.

Applying that definition for input resistance to the semi-finite CIC system, produces an expression which, in addition to structural cable properties, also includes the ratio between the initial potentials (or currents) at the ER lumen and the cytosolic compartment. Accordingly, at identical position and CIC structure, different synaptic signals (i.e. different *I* parameters) are subjected to different input resistance:
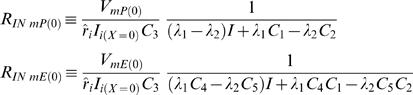
where all the definitions follow the definition given in the main text (see Eq. H13.1–H14.8).

Similarly, the conventional definition of resistance at the *finite* CIC, depends on the ratio between the potentials of ER lumen and the cytosolic compartment at the initial point and at the ending point, as well as the electrotonic length of the specific finite CIC. For simplicity, the calculation of successive CIC systems, in Figure 4, approximated the input resistance at each *finite* CIC system to be determined only by the ratio of the initial potentials. In the interest of completeness we also provide a more detailed expression of the input resistance in a finite CIC system without employing the approximation of the input resistance at each *finite* CIC system by being determined only by the ratio of the initial potentials.

The explicit solution for the steady-state input resistance of finite CIC with arbitrary boundary condition: